# Bioinformatics deciphers the thebaine biosynthesis pathway in opium poppy: Hub genes, network analysis, and miRNA regulation

**DOI:** 10.1016/j.jgeb.2024.100422

**Published:** 2024-08-21

**Authors:** Zahra Shirazi, Mahsa Rostami, Abozar Ghorbani, Pietro Hiram Guzzi

**Affiliations:** a Biotechnology Research Department, Research Institute of Forests and Rangelands, Agricultural Research Education and Extension Organization (AREEO), National Botanical Garden, Tehran Karaj Freeway, P.O. Box 13185-116, Tehran, Iran; b Nuclear Agriculture Research School, Nuclear Science and Technology Research Institute (NSTRI), Karaj, Iran; c Department of Surgical and Medical Sciences, Magna Graecia University of Catanzaro, Catanzaro, Italy

**Keywords:** Analysis, Gene networks, Opium poppy, Pathway, Secondary metabolites, Thebaine

## Abstract

Thebaine, a vital precursor in the codeine and morphine pathway, shows promise in addiction treatment. We conducted a comprehensive study on the thebaine biosynthesis pathway in opium poppy, utilizing bioinformatics tools. The dataset comprising the thirteen genes associated with the thebaine biosynthesis pathway was compiled from an extensive review of published literature and validated using the NCBI BLAST tool. Utilizing STRING and Cytoscape, we analyzed gene interactions and visualized the molecular interaction network, respectively. To identify hub proteins, CytoHubba was administered. The Kyoto Encyclopedia of Genes and Genomes (KEGG) and Gene Ontology (GO) at STRING were used for the enrichment analysis of the hub genes. CytoCluster was used to analyze the network in clusters. Promoter regions of hub genes and potential miRNAs were explored using MEME and the psRNATarget database. Hub genes crucial to thebaine biosynthesis were identified, contributing to essential cellular functions like growth, development, stress response, and signal transduction. Metabolic processes emerged as pivotal for thebaine production, indicating a broader role for the thebaine pathway gene network beyond primary metabolite production. Cell component subnetwork genes demonstrated associations with anatomical units, indicating involvement in plant defense responses. Dominant molecular functions drove plant defense responses. KEGG pathway analysis highlighted the significance of metabolic pathways and biosynthesis of secondary metabolites. Cluster analysis emphasized the relevance of the biosynthesis of amino acids, confirming the link between primary and secondary metabolites. Promoter analysis suggested the potential involvement of signal transduction in thebaine production. Hub genes were targeted by 40 miRNAs, suggesting potential novel biomarkers or target genes within the thebaine biosynthesis pathway. Based on the role of miRNAs identified in connection with the hub genes of the thebaine production process, the secondary metabolite pathway of thebaine appears to be associated with several key plant pathways, e.g. growth, development and stress response. However, these findings, based on bioinformatics analysis, warrant further experimental validation and promise to advance our understanding of the biosynthesis of thebaine and its interactions with other genes and metabolic pathways that influence the production of metabolites.

## 1 Introduction

*Papaver somniferum* L., a member of the Papaveraceae family, stands as a globally significant medicinal plant, renowned for its secondary benzylisoquinoline alkaloids (BIAs), including morphine, codeine, thebaine, and oripavine. These BIAs exhibit therapeutic effects, ranging from pain reduction to addiction treatment and cancer therapy 1, 2, 3. BIAs have the effect of reducing pain, treating addiction and cancer therapy and in improper use, they lead to addiction 4, 5, 6. Opium poppy cultivars exhibit varying BIA compositions, with some rich in morphine and codeine, and others in thebaine and oripavine 7, 8. Thebaine, a pivotal precursor in the codeine and morphine pathway, also holds promise for addiction treatment 9, 10. Current synthetic methods for codeine production involve drawbacks, including the use of organic solvents and toxic agents, emphasizing the need for alternative, more sustainable approaches ^11^. Currently, 85–90 % of the required codeine is obtained synthetically through the methylation of morphine, which is more abundant than codeine in poppy. In this synthesis method, a large number of organic solvents, a toxic methylation agent, and the production of a byproduct of cancer are required, and on the other hand, downstream purification processes are also complicated. Although the natural synthesis of codeine has also been developed using poppy species with the ability to produce more codeine, but this method has climatic problems and requires optimization of extraction from the plant 12, 13. The biosynthesis of plant secondary metabolites is a complex process and is regulated by many genes and also influenced by numerous factors ^14^.

The function of secondary metabolites is the response to a wide range of environmental changes in plants ^15^. Finding the molecular basis of the secondary metabolite biosynthesis and using it to produce secondary metabolites is one of the goals of producers 16, 17. The biosynthesis of BIAs is a complex, gene-regulated process influenced by various factors, and understanding the molecular basis is essential for optimizing production. Thirteen enzymes in the BIAs pathway, particularly related to thebaine biosynthesis, have been identified in the opium poppy ^3^. This includes a sequence of decarboxylations, meta-hydroxylations, and transaminations converting L-Tyrosine to thebaine. Tyrosine aminotransferase (TyrAT) catalyzes the transamination of l-Tyr and produces decarboxylation of 4-hydroxyphenylacetaldehyde, which is the precursor of the group of BIAs ^18^.

With the increase in the number of plant genome sequences and post-genomic research, the basic problem is to use these genomic data for a deeper understanding of the many molecular mechanisms that are the basis for creating complex traits. A gene network shows interactions between genes, where vertices (nodes) of genes and edges (connections) represent interactions between genes which is the application of network analysis in plant biology. Genes connected with direct or indirect interaction are likely to function in the same biological process. Networks represent potential interactions between genes and lead to a systematic understanding of the molecular mechanisms underlying biological processes ^19^. The network is a suitable approach to display a variety of biological data, including protein–protein interactions, gene regulation, cellular pathways, and signal transduction. Identifying and evaluating nodes and their importance in the network leads to understanding and identifying the central elements of biological networks ^20^. One of the approaches in networks is cluster analysis to identify functional modules and predict protein complexes and network biomarkers ^21^.

With the rise of plant genome sequencing, network analysis offers a valuable tool for understanding gene interactions and their role in complex traits. In opium poppy, network analysis has revealed insights into biosynthetic pathways under stress conditions ^22^. This study aims to identify new genes and networks related to the thebaine biosynthesis pathway using databases and software, providing a deeper understanding of the relationships between biosynthetic genes and functional pathways for potential genetic engineering applications, aligning with research and industrial goals.

## 2 Materials and methods

### 2.1 Thebaine pathway genes and protein data

Data on the number and name of genes involved in the thebaine biosynthetic pathway in *P. somniferum* were collected from articles that analyzed the genome of thebaine molecular pathway individually or collectively 3, 23. In these two articles, the identification of the opium poppy genome and the clustering of the genes of the biosynthesis pathway of morphinan have been investigated. Subsequently, the names of 13 genes associated with thebaine biosynthesis were achieved and blasted in NCBI to acquire protein sequences. Then, using the STRING database, 13 obtained protein sequences are used to analyze a protein–protein interaction (PPI) network. The information on 13 genes of theban biosynthesis pathway, including ID genes and annotation, is given in supplementary Table 1.

### 2.2 Protein-protein interaction (PPI) network analysis

The 13 sequences related to thebaine biosynthesis proteins were entered into the STRING (version 10) (https://string-db.org) software to predict their functional interactions with other proteins in *P. somniferum*. The STRING database integrates both known and predicted PPIs for more than 2000 organisms.

An interaction score threshold of > 0.150 was applied to establish the Protein-protein interaction (PPI) network. Cytoscape (version 3.9.1) software was then employed to visualize the PPI network. The CytoHubba plugin (version 0.1) within Cytoscape was utilized to explore the hub proteins between all nodes. Four node ranking methods from Cyto-Hubba, including local-based Degree (Deg), Maximal Clique Centrality (MCC), Maximum Neighborhood Component (MNC), and Density of Maximum Neighborhood Component (DMNC), were employed to calculate the scores of nodes within the network, considering the relationships between nodes and their direct neighbors. Hub genes within the PPI network were selected using the CytoHubba plugin, aiming to identify key nodes with high significance in the network topology ^24^. Subsequently, the identified hub genes were entered into the STRING software to predict a subnetwork and Cytoscape software was then employed to visualize the subnetwork.

### 2.3 Analysis of gene ontology and pathway enrichment in the subnetwork

To uncover the prominent biochemical pathways associated with the hub genes and their subnetwork, the gene accession numbers associated with the subnetwork were entered into the STRING database. The analysis and functional enrichment section, which followed the network visualization, played a crucial role in this investigation. Results covering Molecular Functions (MF), Cellular Components (CC) and Biological Processes (BP) were extracted for gene ontology (GO) categorization ^25^. In addition, KEGG pathway information was obtained using STRING for enrichment analysis. The threshold of statistical significance was set at p < 0.05.

### 2.4 Subnetwork cluster analysis

The subnetwork of hub genes was clustered using CytoCluster (version 2.1.0) with the Identifying Protein Complex Algorithm (IPCA) (with a threshold of 10) for cluster analysis. The KEGG pathways of the top cluster were then obtained from the STRING databases ^21^. The clusters represent a subset of genes that share a similar expression profile, suggesting a potential functional association ^26^.

### 2.5 Promoter motif analysis of hub genes

The 1 kb upstream flanking regions of the hub genes were extracted from Ensembl Plants Web Services (https://plants.ensembl.org). Conserved motifs in the sequences were identified using MEME Suite (version 5.4.1) (meme.nbcr.net/meme/intro.html) with its default parameters, except for thresholds for P and E values of < 0.01, respectively. Conserved motifs in the sequences were identified using the Tomtom tool (https://meme-suite. org/tools/tomtom, version 5.4.1), which identifies known CRE based on the JASPAR CORE 2022 database with its default parameters, except for thresholds for P and E values of <0.01 and <0.0001, respectively ^27^. The GoMo tool (https://meme-suite.org/tools/gomo) was also used to identify possible roles for theme ^28^.

### 2.6 Discovery of miRNAs associated with hub genes

The identification of potential miRNAs linked to hub genes involved an analysis using the psRNATarget database (https://plantgrn.noble.org/psRNATarget/). Parameters, including max expectation 3 and target accessibility (UPE) 25, were applied during the analysis. The prediction of miRNAs associated with hub genes was then performed with the Cytoscape software ^29^.

## 3 Results and discussions

### 3.1 **PPI network** analysis

**Fig. 1:**
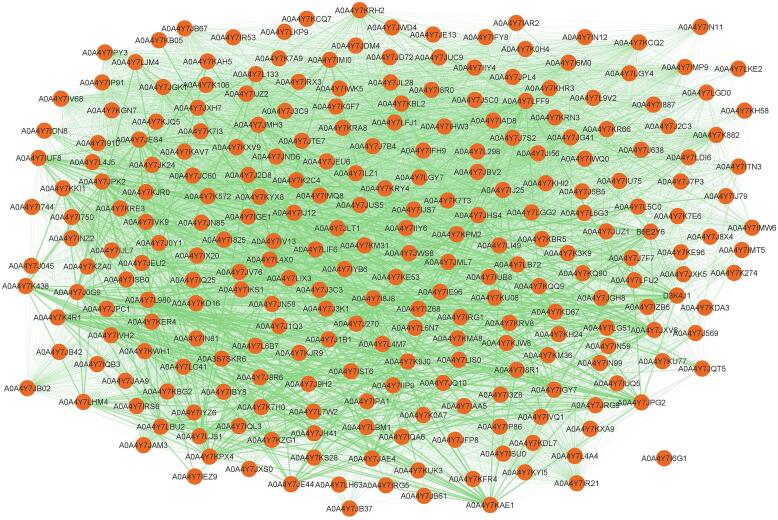
PPI Network of the thebaine biosynthesis genes in opium poppy using Cytoscape software and STRING database.

**Fig. 2:**
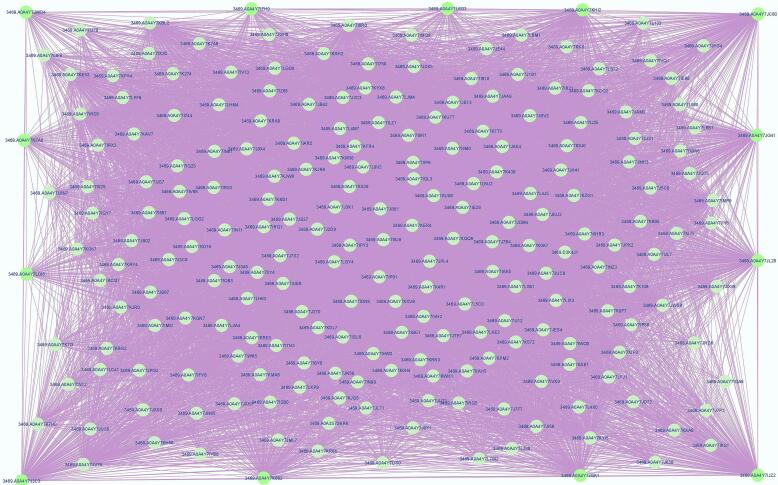
Subnetwork of hub genes in thebaine biosynthesis using the CytoHubba App. 13 hub genes are located on the perimeter of the rectangle.

This study aims to uncover novel genes and networks associated with the thebaine biosynthetic pathway in *P. somniferum* and to provide a better understanding of the connections between biosynthetic genes and functional pathways. The STRING database provided a result with 259 nodes and 9286 edges (Fig. 1) of all interactions. The nodes and edges were transferred to Cytoscape to obtain a PPI network. The CytoHubba analysis section showed 13 hub genes with the most interactions (Table 1). The subnetwork is formed by these identified hub genes and all 223 genes of the sub-network were listed with annotation and ID (Fig. 2. supplementary Table 2). Based on annotation, LOC113276042, LOC113321134, LOC113293394, LOC113317056, LOC113294804 and LOC113309099 were uncharacterized proteins.

**Table 1:** Ranking of hub genes identified in thebaine biosynthesis using CytoHubba computational algorithm, including MNC, MNCC, Degree and MCC.

No	String name	Gene ID		Selected BY	Annotation
1	A0A4Y7JC60	LOC113276042	C5167_006030	MCC	Uncharacterized protein
2	A0A4Y7L6G3	LOC113317056	C5167_042431	DMNC	Uncharacterized protein
3	A0A4Y7JWD4	LOC113287643	C5167_009087	DMNC	PPIase cyclophilin-type domain-containing protein
4	A0A4Y7IJZ2	LOC113339468	C5167_041029	Degree, MNC, MCC	SCP domain-containing protein; Belongs to the CRISP family
5	A0A4Y7J3C3	LOC113356449	C5167_013306	MCC	TPR_REGION domain-containing protein
6	A0A4Y7KZA0	LOC113309099	C5167_001683	DMNC	Uncharacterized protein
7	A0A4Y7JG41	LOC113274034	C5167_006022	Degree, MNC	Thioredoxin; Belongs to the thioredoxin family
8	A0A4Y7LDI6	LOC113321134	C5167_045833	DMNC	Uncharacterized protein
9	A0A4Y7KHI2	LOC113293394	C5167_034724	Degree, MNC, MCC	Uncharacterized protein; Belongs to the cytochrome P450 family
10	A0A4Y7K882	LOC113294804	C5167_031459	MCC	Uncharacterized protein
11	A0A4Y7JGK1	LOC113277294	C5167_021986	Degree, MNC	RNA helicase
12	A0A4Y7JL28	LOC113277422	C5167_022489	Degree, MNC	Thioredoxin; Belongs to the thioredoxin family
13	A0A4Y7IFH9	LOC113353081	C5167_039392	DMNC	PPIase cyclophilin-type domain-containing protein

Two of the hub genes (LOC113277422 and LOC113274034) belong to the thioredoxin family. Thioredoxins are small disulfide redox proteins found in all organisms. They are a general protein disulfide oxidoreductase that interacts with a variety of proteins. Thioredoxins function to regulate the cellular redox environment and are involved intracellularly in a variety of reactions related to metabolism, defense and development. Thioredoxins have several isoforms based on amino acid sequences, which are classified into different groups, subgroups and subcellular localizations such as mitochondria 30, 31, 32. Different pathways reduce thioredoxin in plants, including ferredoxin-thioredoxin reductase, thioredoxin reductase, and the glutathione/glutaredoxin system ^33^. Mitochondrial TRXs are essential for the functioning of metabolic pathways, including stomatal function and antioxidant metabolism under stress conditions. In Arabidopsis, the mitochondrial TRX system regulates primary and secondary metabolism and the plants become more resistant to salt stress ^34^. Recent studies show that TRX enzymes have an important function in aspects of plant immune signaling ^35^.

Two of the hub genes, namely LOC113353081 and LOC113287643, encode for PPIase cyclophilin-type domain-containing proteins. Cyclophilins are widely distributed proteins, many of which have peptidyl-prolyl cis–trans isomerase (PPIase) or rotamase activity ^36^. PPIase activity of cyclophilins is regulated by redox mechanisms and is induced under stress conditions resulting from the redox state. Proteins called theses have multiple functions, localizing in various organs to regulate growth and development processes such as hormone signaling and stress response ^36^. *AtCYP20-3* is an Arabidopsis PPIase that localizes in the chloroplast and is susceptible to photo-oxidation and stress-induced ROS. However, it can be restored by photo-reduced TRX *AtCYP20-3* mutants exhibiting hypersensitivity to oxidative stress due to their involvement in the Cys-based thiol pathway, which modulates light and stress conditions ^37^.

The hub gene, LOC113339468, contains an SCP domain, making it a member of the CRISP family. The SCP region is also known as the CAP region. The broader family of SCP-containing proteins includes plant pathogenesis-related protein 1 (PR-1), CRISPs, mammalian cysteine-rich secreted proteins that combine SCP with a C-terminal cysteine-rich domain, and vespid venom-derived allergen suggested that SCP regions are capable of functioning as endopeptidases. Within the concavity of the CAP1/PR1 domain, members of the CAP family perform diverse physiological functions through binding to small molecules and proteins. To prevent the multiplication of pathogens, the plant PR1 protein and the yeast CAP proteins Pry1 and Pry2 bind sterols and lipids. Bacteria and eukaryotes rely on sterols, and their growth is impeded when they are removed from the membranes of a pathogen or when they are killed as a result ^38^.

The LOC113356449 hub gene has been determined to be a protein containing the TPR_REGION domain. The tetratricopeptide repeat (TPR) is present in numerous proteins found in both prokaryotic and eukaryotic organisms. TPR motifs offer an efficient module that assists in the construction of diverse protein complexes and often has a significant role in essential cell processes by providing TPR-containing proteins ^39^. TPR plays a crucial role in several proteins involved in diverse biological processes such as gene regulation, mitosis, steroid receptor function, and protein import ^40^. Proteins containing TPR domains have gained importance in plant hormone signaling. Additionally, TPR proteins have also shown their involvement in gibberellin, cytokinin, and auxin responses, as well as in the production of ethylene 41, 42.

The LOC113293394 hub gene belongs to the cytochrome P450 family. The cytochrome P450 superfamily, which is also found in mammals, fungi, bacteria and many other organisms, is the most extensive protein family of plant enzymes. The members of such a family are part of many metabolic pathways, each with different and complex functions that play an important role in a variety of reactions. Therefore, many primary metabolites are synthesized to play a role in growth and development signals or to protect plants against a variety of bacterial and auxiliary stresses ^43^. Cytochrome P450s (CYPs) are the largest enzyme family involved in NADPH- and/or O_2_-dependent hydroxylation reactions in all domains of life. CYPs play a very important role in the elimination of xenobiotics from plants and animals. In addition to this function, CYPs act as catalysts and play an important role in the synthesis of other metabolites, antioxidants, or phytohormones from high plants. New findings have emerged through the use of Next Generation Sequencing, offering fresh insight into the function of CYPs in specific plant processes, such as those related to stress responses. When exposed to environmental stress, gene expression of certain CYPs is regulated, influencing the interaction between abiotic and biotic stress responses ^44^.

The characterization of RNA helicase was observed in LOC113277294. RNA helicases (RH) are present in every cell and alter RNA structures and modify ribonucleoprotein complexes through the use of ATP hydrolysis. RHs are involved in several RNA processing and metabolic processes, including gene transcriptional regulation, preRNA splicing, miRNA biogenesis, liquid phase separation, or RNA biogenesis, among other molecular processes. Through these mechanisms, root hairs facilitate vegetative and reproductive growth as well as response to abiotic and biotic stress throughout the plant's lifespan ^45^.

The network analysis of genes and pathways associated with thebaine biosynthesis in *P. somniferum* has unveiled a repertoire of key hub genes with multifaceted functions. As mentioned, these encompass uncharacterized proteins, two members of the thioredoxin family, two PPIase cyclophilin-type domain-containing proteins, a SCP domain-containing protein, a TPR_REGION domain-containing protein, and a cytochrome P450 family member, alongside an RNA helicase. These discoveries illuminate the expansive molecular functions and pathways that may play pivotal roles in shaping the thebaine biosynthesis pathway in opium poppy. The identification of these hub genes, implicated in redox regulation, stress response, and various biological processes, accentuates their potential significance in orchestrating the intricate dynamics of the thebaine biosynthesis pathway.

### 3.2 Exploration of gene ontology and pathway enrichment for subnetwork genes in *P. somniferum* associated with thebaine production

**Fig. 3:**
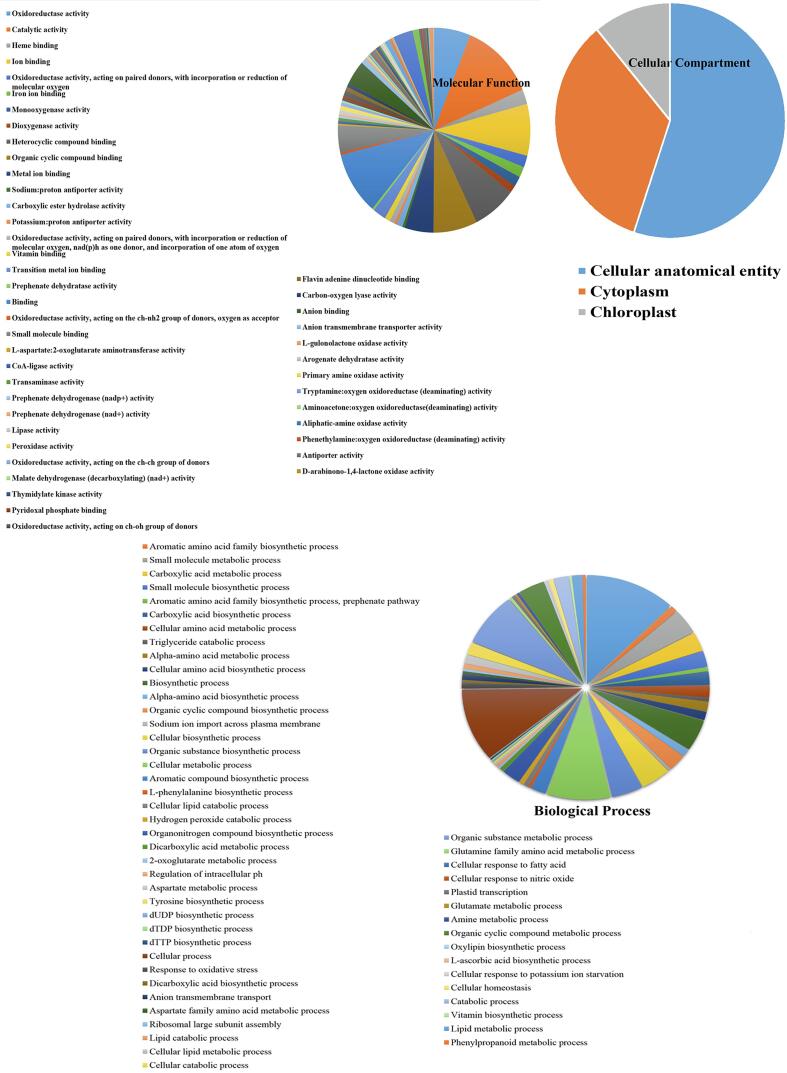
Gene ontology analysis classified the subnetwork nodes into 3 groups: molecular function, biological process, and cellular component.

GO analysis is a well-known method for identifying genes and gene products and representative biological aspects from high-throughput genomic or transcriptomic data, including molecular function (MF), cellular components (CC), and biological process (BP) 46, 47. The results of GO analysis and pathway enrichment are presented (Fig. 3). The dominant (≥50 % identified genes) GO terms found for BP are significantly enriched in metabolic process, cellular process, organic matter metabolic process, and cellular metabolic process. Our results showed that the metabolic process plays an important role in the production of thebaine. Biological activities including specific interactions with ligands or structures of gene products shall be considered to constitute the molecule functions of a gene product. The biochemical activity of the gene product shall be considered as a molecule function which includes specific interaction with ligands and their structures. The ability that a gene product or a gene product complex has as a potential is also covered by this definition ^48^. A closer look reveals that the gene network governing the thebaine pathway extends its influence beyond the well-established metabolic processes associated with secondary metabolite production. While the primary function of the thebaine pathway is acknowledged, the sub-hub gene network emerges as a multifunctional player, participating in diverse biological activities. This suggests that the subnetwork not only contributes to the primary metabolic pathway but also engages in additional processes, indicating a broader functional spectrum. These findings underscore the versatility and complexity of the gene network linked to the thebaine biosynthesis pathway, offering valuable insights into its potential regulatory mechanisms and broader biological implications.

Within the Cellular Components category, the predominant GO terms, representing over 55 % (≥55 % of identified genes) of the identified genes, underscore a distinct association with anatomical units. This implies a specific connection between the genes within the subnetwork and cellular structures integral to the synthesis and regulation of thebaine. The pronounced enrichment in anatomical units suggests that the subnetwork genes actively participate in shaping the cellular environment, possibly contributing to the specialized compartments crucial for thebaine biosynthesis.

Shifting focus to Molecular Function, the dominant GO terms, encompassing over 50 % (≥50 % identified genes) of the identified genes, exhibit significant enrichment in catalytic activity, ion binding, binding, oxidoreductase activity, and heterocyclic compound binding. These molecular functions align with critical processes implicated in the thebaine biosynthesis pathway. Catalytic activity suggests enzymatic involvement, potentially crucial for key biochemical transformations leading to thebaine synthesis. Ion binding and oxidoreductase activity further emphasize the subnetwork genes' potential roles in mediating redox reactions or interacting with ions integral to the thebaine biosynthesis process. Heterocyclic compound binding, a characteristic feature in the MF analysis, hints at the intricate molecular interactions governing the synthesis of thebaine, a heterocyclic alkaloid. Nucleotide binding, ion binding, hydrolase activity, and heterocyclic compound binding are functions involved in the plant's defense response 49, 50.

**Fig. 4:**
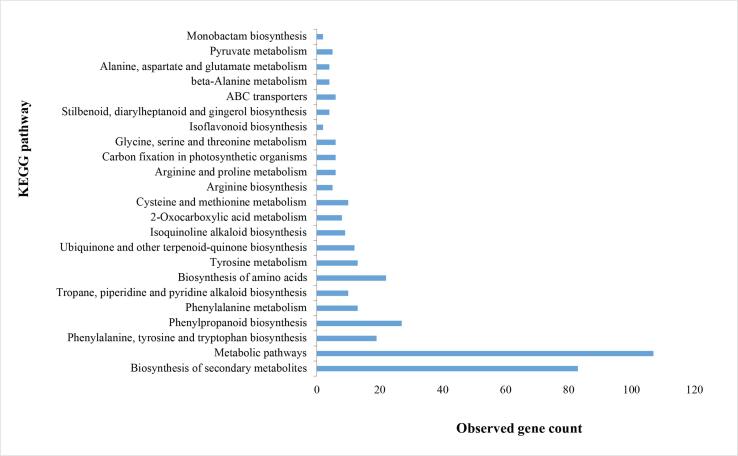
Kyoto Encyclopedia of Genes and Genomes (KEGG) pathways analysis on the hub genes in thebaine biosynthesis using STRING version10.

In conjunction with the Gene Ontology (GO) analysis, a comprehensive understanding of the subnetwork's association with thebaine biosynthesis in *P. somniferum* is further enriched through KEGG pathway enrichment analysis (Fig. 4). This analysis delves into specific pathways that are significantly influenced by the identified subnetwork genes, providing crucial insights into the functional landscape related to thebaine production. The KEGG pathway enrichment analysis reveals that the most pivotal pathways, incorporating over 45 % of the identified genes, are centered on metabolic pathways and the biosynthesis of secondary metabolites. This finding aligns seamlessly with the known and primary function of the thebaine biosynthesis pathway. Metabolic pathways, as a dominant enriched category, indicate the active involvement of the subnetwork genes in a cascade of biochemical reactions crucial for thebaine production. The biosynthesis of secondary metabolites, in particular, holds direct relevance to the production of thebaine, as it represents a key branch of the metabolic network associated with the synthesis of specialized compounds ^51^. These enriched pathways directly substantiate the subnetwork genes' functional significance in driving the thebaine biosynthesis pathway. The prominence of metabolic pathways and biosynthesis of secondary metabolites reinforces the pivotal roles played by the identified genes in orchestrating the complex network of reactions leading to the production of thebaine. This integrated analysis of GO terms and KEGG pathways provides a comprehensive overview of the subnetwork's functional repertoire, affirming its central role in the intricate processes governing thebaine biosynthesis in opium poppy ^52^.

### 3.3 Cluster analysis of the sub-network

Cluster analysis of biological networks is an important strategy for identifying functional modules and predicting network biomarkers and protein complexes. In addition, the structure of the biological networks can be revealed when clustering results are visible. CytoCluster, used in this study, includes six clustering algorithms. It depends on which algorithm is chosen for clustering. The dense subgraphs on the protein interaction networks were represented by the IPCA algorithm, a density clustering algorithm. The weight per edge is determined by identifying the common neighbors of both connected nodes. The sum of the weights of the surrounding edges determines the weight of each node. This weight determines the seed from which a cluster is initially formed. Subsequently, IPCA recursively adds nodes from its neighbors based on their priority. Two prerequisites for adding nodes to a cluster are the interaction probability of a node and the shortest path 1 between each node in that cluster ^21^. In this study, subnetwork cluster analysis identified clusters ranking 1 to 3 (Table 2). Eighteen pathways were common to all three clusters. However, the pathways involved in carbon fixation in photosynthetic organisms, isoflavonoid biosynthesis, and pyruvate metabolism are only represented in cluster rank 2. The analysis revealed that five pathways (2-Oxocarboxylic acid metabolism, ABC transporters, Alanine, Aspartate, and Glutamate metabolism, Arginine and Proline metabolism, and Biosynthesis of amino acids) are the primary pathways in the thebaine pathway, as demonstrated by the overlap of genes counted in KEGG pathways and the results of the cluster analysis. The crucial role of transporters in the transportation of plant secondary metabolites has been validated ^53^. However, amino acids can be regarded as secondary metabolites when produced from primary metabolites ^54^. The aromatic amino acids that are used for protein synthesis and are precursors of many natural products, such as pigments, alkaloids, hormones, and cell wall components, are tryptophan, phenylalanine, and tyrosine. The shikimate pathway is the source of all three amino acids ^55^. Alkaloids are a class of plant secondary metabolites that, until now, have been considered to be basic compounds derived from amino acids containing at least one heterocyclic nitrogen atom such as phenylalanine, tyrosine, and tryptophan. Alkaloids are synthesized via decarboxylation of tyrosine and tryptophan, catalyzed by tyrosine decarboxylase and tryptophan decarboxylase enzymes ^56^. Phenylethylamine alkaloids, simple tetrahydroisoquinoline alkaloids, and modified benzyl tetrahydroisoquinoline alkaloids are alkaloids derived from tyrosine ^57^. The isoquinoline alkaloids are tyrosine-derived plant alkaloids with a skeleton of isoquinolines. An important group consists of benzylisoquinoline alkaloids. The alkaloids of isoquinoline, which are the benzene ring fused to the pyridine ring, contain thebaine ^1^. Consequently, secondary metabolites or natural substances are a diverse set of naturally occurring metabolic products that are not essential for the vegetative growth of the producing organisms. For instance, they are perceived as components that promote differentiation by acting as defense elements or signal molecules in environmental interactions, symbiosis, metal transport, and competition, among other things. These chemicals are known to have an adaptive function.^58^.

**Table 2:** Summary of clusters (rank 1 to 3) resulting from cluster analysis of the subnetwork of hub genes in thebaine biosynthesis using the CytoCluster app.

Cluster Rank	Nodes	Edges	Pathways	
1	110	4236	beta-Alanine metabolism Biosynthesis of secondary metabolites Glycine, serine and threonine metabolism Flavonoid biosynthesis Lysine biosynthesis	2-Oxocarboxylic acid metabolism ABC transporters Alanine, aspartate and glutamate metabolism Arginine and proline metabolism Arginine biosynthesis Biosynthesis of amino acids Biosynthesis of secondary metabolites Cysteine and methionine metabolism Isoquinoline alkaloid biosynthesis Metabolic pathways Monobactam biosynthesis Phenylalanine metabolism Phenylalanine, tyrosine and tryptophan biosynthesis Phenylpropanoid biosynthesis Stilbenoid, diarylheptanoid and gingerol biosynthesis Tyrosine metabolism Ubiquinone and other terpenoid-quinone biosynthesis Tropane, piperidine and pyridine alkaloid biosynthesis
2	108	4151	beta-Alanine metabolism Biosynthesis of secondary metabolites Carbon fixation in photosynthetic organisms Glycine, serine and threonine metabolism Isoflavonoid biosynthesis Pyruvate metabolism	
3	108	4128	Flavonoid biosynthesis Lysine biosynthesis	

### 3.4 Promoter motif analysis of hub genes

The conserved motifs and consensus *cis*-regulatory elements (CREs) of hub genes were analyzed through the UFRs, which were discovered at Ensemble Plants. MEME identified eight major motifs in the promoter gene. Furthermore, GOMo analysis indicated that the transcription factor motifs, including BP, MF and CC, had several associated biological functions. The CREs were found to regulate transcription, DNA-dependent and responses to ethylene as revealed by GO. These are the CREs that took part in MF, including transcription factor and receptor activity. Additionally, the majority of significant GO terms for CC were enriched in the endomembrane system and plasma membrane (Table 3).

**Table 3:** The conserved motifs found in promoters of the hub gene by MEME analysis.

**Motif**	**Logo**	**Width**	**Top 5 specific prediction**
MA1873.1		5	MF transcription factor activity BP regulation of transcription, DNA-dependent CC plasma membrane
MA1883.1		3	MF transcription factor activity CC plasma membrane
MA1895.1		3	MF transcription factor activity BP regulation of transcription CC endomembrane system
MA18.96.1		4	MF transcription factor activity BP regulation of transcription CC endomembrane system
MA1900.1		3	MF transcription factor activity CC endomembrane system
MA1907.1		5	MF transcription factor activity BP regulation of transcription, response to ethylene stimulus CC endomembrane system
MA1924.1		4	MF transcription factor activity BP regulation of transcription CC endomembrane system
MA19025.1		5	MF transcription factor activity, receptor activity BP regulation of transcription CC endomembrane system

The study identified the key processes by which TFs participate in the biosynthesis of thebaine as responses to ethylene. The internal membrane structure comprises important components of the endoplasmic reticulum, Golgi, vesicles, cell membranes, and nuclear envelope that interact among themselves within cells. Triggered by stress and hormones, the membrane receptors initiate a signal transduction cascade. The inner membrane system is likely to facilitate signal transduction and enhance the synthesis of metabolites like thebaine in response to hormones. Nonetheless, additional research is recommended to investigate the correlation between these genes and CREs in thebaine production ^59^.

### 3.5 Identification of miRNAs that target hub genes

**Fig. 5:**
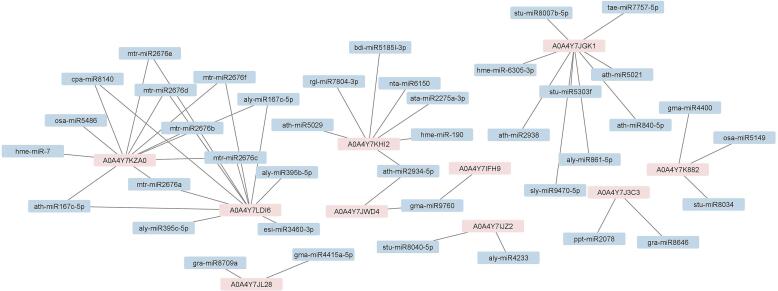
Prediction of potential miRNAs targeting the hub genes in thebaine biosynthesis using psRNATarget.

MicroRNAs, a family of endogenous small-noncoding RNAs, regulate gene expression in most eukaryotes ^60^. One of the objectives of the current study was to identify miRNAs that can target the hub genes. Thus, possible miRNAs that target hub genes have been predicted using a psRNATarget program, a total of 40 miRNAs were found for 10 genes out of 13 hub genes (Fig. 5). The hub genes were targeted by more than one miRNA. The gma-miR9760 was targeted A0A4Y7JWD4 and A0A4Y7IFH9 hub genes, while mtr-miR2676a, mtr-miR2676b, mtr-miR2676c, mtr-miR2676d, mtr-miR2676e, mtr-miR2676f, cpa-miR8140, ath-miR167c-5p, aly-miR167c-5p and ath-miR167c-5p were common for A0A4Y7KZA0 and A0A4Y7LD16. One of the most conserved miRNA families in plants is miR167. It participates in regulating the development of roots, stems, leaves and flowers, flowering time, embryonic development, seed development and stress response by regulating *auxin response factors (ARFs)* and *IAA resistant3 (IAR3)* genes ^61^. MiR167 plays a major role in the proper patterning of gene expression and reproductive capacity for both ovules and anthers in Arabidopsis ^62^. In the case of soybean nodulation and lateral root development, miR167-directed regulation of auxin response factors is required ^63^. Overexpression of Arabidopsis microRNA167 actuates salicylic acid-dependent defense against *Pseudomonas syringae* through the control of its targets ARF6 and ARF8 ^64^. A major regulator of sulfate metabolism is MiR395. In Poplar, studies have shown the role of MIR395 in the regulation of secondary xylem development by sulfate metabolism ^65^. MiR4233 proposed to be involved in the regulation of expression of the *P5CS* gene, one of the genes responsible for proline synthesis, as part of a study examining the effects of salicylic acid use on miRNA expression in wheat cultivars under drought stress ^66^. It is clear that the secondary metabolite production pathway of thebaine appears to have an association with a number of key plant pathways, e.g. growth, development and stress response, based on the role of miRNAs identified in connection with hub genes for the thebaine production process.

### 3.6 Proposed pathway and the role of key genes in thebaine biosynthesis

According to the signaling function of hub genes, these genes are probably located upstream of the genes involved in the biosynthesis of thebaine. Their encoded proteins are activated in response to internal and external signals received by the plant, including biotic and abiotic stresses, and activate a cascade of downstream signaling pathways.

The analysis of sub-network genes related to hub genes also shows that after the activation of the signaling pathway by the hub genes, the next pathways include the activation of the genes for the production of primary metabolites, including the genes of the shikimate pathway. These pathways are involved in the production of precursors of thebaine biosynthesis and other metabolites, including tyrosine. Analysis of the promoter motif of hub genes showed conserved motifs related to transcriptional activity and their regulation of transcription factors and their association with the membrane. Identified miRNA also controls post-transcriptional regulatory mechanisms of hub genes in response to signal reception. These results, like the results obtained from the activity of hub genes, confirm the role of these genes in signal reception and the initiation of signaling responses by transcription factors and the effect on activating the primary and secondary response pathways of the plant. Finally, the plant responds to receiving abiotic and biotic signals by producing secondary metabolites, including thebaine.

## 4 Conclusion

In conclusion, this study sheds light on the intricate molecular landscape governing thebaine biosynthesis in *P. somniferum*. The protein–protein interaction (PPI) network analysis, coupled with the identification of hub genes, has unraveled a diverse set of players involved in redox regulation, stress response, and metabolic pathways. The inclusion of both characterized and uncharacterized proteins within the subnetwork underscores the complexity of the thebaine biosynthetic pathway. The functional characterization of hub genes, such as those belonging to the thioredoxin family, PPIase cyclophilin-type domain-containing proteins, SCP domain-containing proteins, TPR_REGION domain-containing proteins, and cytochrome P450 family members, alongside an RNA helicase, highlights their multi-faceted roles. These genes contribute to crucial processes ranging from redox regulation and stress response to diverse biological activities, underlining their significance in shaping the dynamics of the thebaine biosynthesis pathway. The gene ontology and pathway enrichment analyses provide a comprehensive overview of the subnetwork's functional repertoire. Dominant terms in biological processes, cellular components, and molecular functions underscore the active involvement of subnetwork genes in metabolic processes crucial for thebaine production. KEGG pathway enrichment analysis further solidifies the connection, emphasizing the central roles played by these genes in metabolic pathways and the biosynthesis of secondary metabolites. Cluster analysis reveals shared pathways among hub genes, offering insights into the coordinated regulation of processes like 2-Oxocarboxylic acid metabolism, ABC transporters, and amino acid biosynthesis. This interconnectedness emphasizes the subnetwork's holistic contribution to the thebaine biosynthesis pathway. These findings hint at a complex regulatory network orchestrating thebaine biosynthesis, where transcriptional and post-transcriptional regulation collaborate to fine-tune the expression of key genes. This study significantly advances our understanding of the molecular intricacies underlying thebaine biosynthesis. The identified hub genes, enriched pathways, and regulatory elements provide a comprehensive framework for future investigations and genetic engineering endeavors aimed at optimizing the production of thebaine and related alkaloids in opium poppy. The multifaceted roles of these genes open avenues for targeted interventions, offering the potential to enhance alkaloid yields and explore the broader biological implications of the thebaine biosynthesis pathway.

## CRediT authorship contribution statement

**Zahra Shirazi:** Writing – original draft, Methodology, Investigation, Data curation. **Mahsa Rostami:** Writing – review & editing, Visualization, Software. **Abozar Ghorbani:** Writing – review & editing, Validation, Supervision, Software, Methodology, Formal analysis, Conceptualization. **Pietro Hiram Guzzi:** Writing – review & editing.

## Declaration of competing interest

The authors declare that they have no known competing financial interests or personal relationships that could have appeared to influence the work reported in this paper.
