# Supplementary material for: Bioinformatics deciphers the thebaine biosynthesis pathway in opium poppy: Hub genes, network analysis, and miRNA regulation
Source: J Genet Eng Biotechnol. 2024 Aug 21;22(4):100422. doi: 10.1016/j.jgeb.2024.100422 (PMC11387676; doi:10.1016/j.jgeb.2024.100422)
Supplement: Supplementary Data 1 [file mmc1.docx]

| Gene Name | Ensemble ID | String ID | Annotation |
| --- | --- | --- | --- |
| TyrDC | C5167_008148 | A0A3S7SKR6 | Tyrosine decarboxylase |
| TyrAT | C5167_039575 | D3K4J1 | Tyrosine aminotransferase |
| NCS | C5167_036379 | A0A4Y7I6G1 | Bet_v_1 domain-containing protein |
| 6OMT | C5167_025088 | A0A4Y7JTE7 | Uncharacterized protein; Belongs to the class I-like SAM-binding methyltransferase superfamily. Cation-independent O-methyltransferase family. |
| CNMT | C5167_031428 | A0A4Y7K7I3 | Uncharacterized protein |
| NMCH (CYP80B3) | C5167_025060 | A0A4Y7JRG9 | MACPF domain-containing protein |
| 4′OMT2 | C5167_031357 | A0A4Y7K7A9 | Uncharacterized protein; Belongs to the class I-like SAM-binding methyltransferase superfamily. Cation-independent O-methyltransferase family. |
| BBE | C5167_043609 | A0A4Y7L980 | FAD-binding PCMH-type domain-containing protein; Belongs to the oxygen-dependent FAD-linked oxidoreductase family. |
| REPI (CYP82Y2) | C5167_047531 | A0A4Y7LIF6 | Aldo_ket_red domain-containing protein |
| SalSyn (CYP719B1) | C5167_047533 | A0A4Y7LKP9 | Uncharacterized protein |
| SalR | C5167_047536 | A0A4Y7LGY4 | Uncharacterized protein; Belongs to the short-chain dehydrogenases/reductases (SDR) family. |
| SalAT | C5167_047541 | B6E2Y6 | Salutaridinol 7-O-acetyltransferase |
| THS | C5167_047537 | A0A4Y7LGY7 | Bet_v_1 domain-containing protein |

Supplementary Table 1. The information of 13 genes of the biosynthetic pathway of thebain along with ensemble and string IDs and their annotation.
